# A Noninvasive 3D Body Scanner and Software Tool towards Analysis of Scoliosis

**DOI:** 10.1155/2019/4715720

**Published:** 2019-05-09

**Authors:** Susmita Roy, Alexander T. D. Grünwald, Ana Alves-Pinto, Robert Maier, Daniel Cremers, Daniela Pfeiffer, Renée Lampe

**Affiliations:** ^1^Technical University of Munich, School of Medicine, Klinikum rechts der Isar, Orthopaedic Department, Research Unit of the Buhl-Strohmaier Foundation for Cerebral Palsy and Paediatric Neuroorthopaedics, Munich, Germany; ^2^Technical University of Munich, Computer Vision Group, Department of Computer Science, Munich, Germany; ^3^Technical University of Munich, School of Medicine, Klinikum rechts der Isar, Department of Diagnostic and Interventional Radiology, Munich, Germany; ^4^Markus Würth Professorship, Technical University of Munich, Munich, Germany

## Abstract

**Purpose:**

Children with neurological disorders, such as cerebral palsy (CP), have a high risk of developing scoliosis during growth. The fast progression of scoliosis implies in several cases frequent clinical and X-ray examinations. We present an ionizing radiation-free, noncontacting method to estimate the trajectory of the vertebral column and to potentially facilitate medical diagnosis in cases where an X-ray examination is not indicated.

**Methods:**

A body scanner and corresponding analysis software tools have been developed to get 3D surface scans of patient torsos and to analyze their spinal curvatures. The trajectory of the vertebral column has been deduced from the body contours at different transverse sectional planes along the vertical torso axis. In order to verify the present methods, we have analyzed twenty-five torso contours, extracted from computer tomography (CT) images of patients who had a CT scan for other medical reasons, but incidentally also showed a scoliosis. The software tools therefore process data from the body scanner as well as X-ray or CT images.

**Results:**

The methods presented show good results in the estimations of the lateral deviation of the spine for mild and moderate scoliosis. The partial mismatch for severe cases is associated with a less accurate estimation of the rotation of the vertebrae around the vertical body axis in these cases. In addition, distinct torso contour shapes, in the transverse sections, have been characterized according to the severity of the scoliosis.

**Conclusion:**

The hardware and software tools are a first step towards an ionizing radiation-free analysis of progression of scoliosis. However, further improvements of the analysis methods and tests on a larger number of data sets with diverse types of scoliosis are necessary, before its introduction into clinical application as a supplementary tool to conventional examinations.

## 1. Introduction

Scoliosis is a 3D spinal deformity characterized by a lateral deviation of the spine of at least 10° in the frontal plane and in a vertebral rotation [1, 2]. One of the measures, in radiography, used to quantify the curvature of scoliosis is the Cobb angle [3]. This is defined as the included angle between the upper and lower end plates of the most tilted vertebrae around the apex of the spinal curvature in frontal view [1, 3–5]. The Cobb angle is thus indicative of the severity of the scoliosis, which can be classified accordingly as (a) mild, when characterized by a small spinal deformity with a Cobb angle in the range 10-20° and generally managed with physiotherapy; (b) moderate, when the Cobb angle is in the range 20-40° and generally requiring the use of a brace; and (c) severe, when the Cobb angle exceeds 40° and surgical intervention is often needed [1]. Besides the lateral deviation of the vertebral column, a scoliosis (often) includes a rotation of the vertebrae around the vertical axis, which results in an asymmetric torso contour from a thoracic prominens and/or a rib hump, due to the articulation of the vertebral bodies with the ribs. Other clinically observable signs of scoliosis are a difference in shoulder height, a pelvic discrepancy and differences in torso shape.

Scoliosis can be furthermore classified according to its etiology, as either idiopathic or neurogenic. A scoliosis is* idiopathic* when an etiology cannot be specified and the X-ray shows a rotation of the vertebral body within the curve [1, 2]. This form can be further subdivided into (a) infantile, if it is diagnosed before the age of three, (b) juvenile, if diagnosis occurs between three and ten years of age; and (c) adolescent, when diagnosed beyond the age of ten and until the end of growth [1]. The latter type sums up to more than 80 % of cases, while juvenile and infantile idiopathic scoliosis account for the rest [6]. Idiopathic scoliosis show often* S*-shaped curves.

A scoliosis is* neurogenic* when there are neuromuscular conditions involved in its etiology, such as cerebral palsy (CP). Patients affected by CP develop commonly a neurogenic scoliosis at an early age, with a strong correlation between the severity of motor deficits and the rate of progression of scoliosis being observed, due to their typical muscular imbalances and spastic muscle tone. In comparison to idiopathic scoliosis, neurogenic scoliotic curves exhibit often a large-bowed* C*-shape. Severe neuromuscular scoliosis affects the balance and sitting abilities of patients and can lead to back and rib pain, as well as cardiac and lung complications, that add difficulties to daily care [7, 8].

Diagnosis of scoliosis during growth entails in general a higher risk of progression and aggravation of the deformations. It is however difficult to predict how scoliosis progresses over time. In particular children showing first indications of an evolving scoliosis or a malposition of their vertebrae undergo therefore frequent clinical and radiography examinations, since therapy is likely to be more effective with an early diagnosis [9]. Neurogenic scoliosis in particular exhibit often a fast progression and thus may require monitoring at short intervals [7, 8].

As a consequence of frequent radiography examinations, and despite the development and use of modern low dose X-ray equipment [10], young patients showing first indications of a scoliosis are often exposed to significant ionizing radiation and have a higher risk of radiation-related health problems.

A significant effort thus has been made to develop ionizing radiation-free, noninvasive and reliable imaging methods for the diagnostic and analysis of spinal deformities, in order to reduce the exposure to ionizing radiation during follow-ups, in particular for children and adolescents. These ionizing radiation-free methods are based on Moiré fringes [11, 12], rasterstereography [13] and 3D optical methods, such as the integrated shape imaging system (ISIS) [14, 15], or laser triangulation [16]. However, some of these methods require long preparation times with patients. Physically impaired people might be therefore overstrained by these examining procedures.

The purpose of the current study was to find an analysis method, using an ionizing radiation-free, fast and noncontacting body scanner, to (a) estimate the trajectory of the vertebral column and the rotation of the vertebral body with respect to their spinous processes from a 3D surface scan and (b) to derive the lateral deviation of the spine similar to that obtained from an X-ray image.

The methods here presented have been developed with the perspective of a future clinical application: in case a patient, in his first consultation with an orthopedic specialist, presents signs of scoliosis, a 3D surface scan of the torso will be done with the body scanner, in addition to the regular X-ray image. Deviations of the spine will be not only obtained from the X-ray, but will also be estimated by analyzing the body scanner 3D surface scan with the analysis methods described here. Later in the following examination, in addition to the clinical examination, a 3D surface scan will be captured with the body scanner, from which the trajectory of the spine will be calculated. If this is not remarkably different from that obtained in the first consultation, this may help to decide whether another X-ray is indicated, or not, at that stage. Given that the methods here proposed are to be employed, in the future, to compare the information from 3D surface scans with the initial reference X-ray, the analysis software was conceptualized to handle not only 3D surface scans, but also X-ray and CT scans.

The paper is organized as follows:

- Section 2 of the paper gives an overview of the methods used (Section 2.2): of the body scanner (Section 2.2.1), to collect and analyze the body scanner data (Sections 2.2.2 and 2.2.3) and of the developed software tools (Section 2.2.4).
- Section 3 presents the analysis performed to verify and validate the methods described in Section 2. This verification was done by applying the methods developed to a number of torso contours extracted from available computer tomography (CT) data, showing different degrees of spinal curvatures. The results of the analysis methods, essentially scoliosis characteristic like the lateral deviation of the spine, have been successfully verified directly from the different views of the CT images.
- Section 4 describes additional results.

## 2. The 3D Body Scanner and the Torso Shape Analysis Methods

### 2.1. Motivation

The goal was to verify that the body scanner can be used in the clinical practice, especially to examine patients with CP and neurogenic scoliosis, since many of them cannot stand for a long time. The questions were therefore (a) do patients feel comfortable with the scanning procedure? (b) Is the scanning procedure fast enough and does it capture clear images of patient's torso, despite their reduced ability to hold their posture and/or to sit straight without an arm- or backrest?

An additional aim was to develop an analysis method that can give a realistic and reasonable estimate of the deviation of the spine from the 3D surface scan of the torso output by the scanner. If possible, this opens up the possibility of employing a supplementary nonionizing radiation method to support clinical examination of scoliosis. The analysis tools and associated software were therefore developed to extract the spinal deviation from the data collected with the body scanner, an X-ray image and a CT scan image.

### 2.2. Methods

#### 2.2.1. Description of the 3D Body Scanner

**Figure 1:**
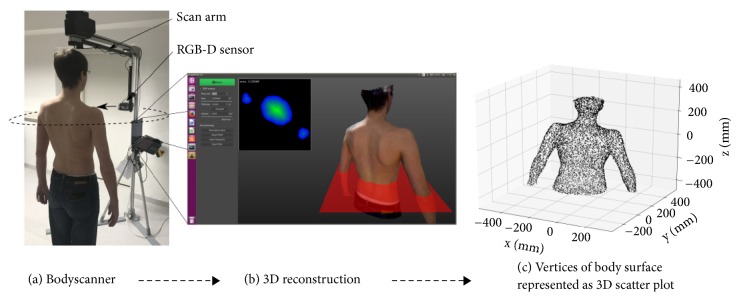
A 3D scan of a person is acquired with a fully automatic body scanner setup. (a) Picture of the body scanner setup. (b) We reconstruct a dense 3D surface scan from the captured RGB-D data, with 4% of its original vertices visualized in (c) for illustration.

The body scanner was designed and developed at the Chair for Computer Vision & Artificial Intelligence in the Faculty of Informatics at the Technical University of Munich. At the core of the body scanner setup, an ASUS Xtion Pro Live RGB-D sensor, similar to the Kinect system used in game consoles, is mounted on a scan arm that rotates in the transverse plane around the person, as shown in Figure 1(a). The RGB-D sensor consists of a color sensor for capturing RGB images and a depth sensor to simultaneously obtain depth measurements of a scene using an infrared laser projector and an infrared camera. During scanning the swivel arm rotates 360° around the patient in the center in about seven seconds, with a diameter of ~1.4 m. The patient stands, or sits, at the rotation center with arms slightly stretched out. The color images and depth profiles with a resolution of 640 × 480 pixels are captured at a real-time frame rate of 30 Hz and subsequently processed with a software package developed specifically for this end, running on a laptop. The total time of scanning and reconstructions sums up to about fifteen seconds. The reconstruction of a conformal 3D surface scan of the patient (Figure 1(b)), which has absolute metric scale, employs a truncated signed distance function as its underlying volumetric representation for surface fusion [17, 18]. The obtained 3D triangle mesh is then exported in a polygon file format with about 150k to 200k vertices, representing the body surface for further processing (Figure 1(c)). The resulting surface accuracy depends on the following aspects: the distance between the sensor and the body surface, the angular velocity at which the sensor is rotating around the human body, the movements of the human body itself while scanning, and the surface structure. The depth accuracy of a single (relatively noisy) depth map is typically between 0.3 and 1.0 cm at a distance of 1.0 m, where the uncertainty increases quadratically with distance. However, fusing several frames using a weighted average effectively reduces the noise and significantly improves the reconstruction quality. In practice, the resulting 3D surface scan exhibits a clean and smooth surface, with an estimated resolution of approximately less than 1.0 mm. The angular velocity of the sensor and the inherent movements of the human body have counter effects on the scan quality. While a fast scan is affected less by movements of the human body, this inevitably leads to higher motion blur and less data for surface fusion. The quality of a slow scan, however, is more prone to potential movements of the human body. The scanning velocity thus needs to be adapted according to the abilities of the patient to stand still and keep her/his posture. In practice, scanning times of about seven seconds turned out to provide good results. Despite the lack of an active motion compensation method in the algorithm, the latter is sufficiently robust against small motions of the scanned person, as movements are smoothed out in the fusion process.

Since the scans are collected with an optical system, the scanning procedure is ionizing radiation-free, noncontacting, and fast. The scanner itself is lightweight, easy to set up, and thus very mobile.

#### 2.2.2. Collection of 3D Surface Scans

At first the body scanner was tested on members of the laboratory. Then 3D surface scans were done (cp. Section 2.2.1) on seventeen patients with idiopathic and neurogenic types of scoliosis and of different degrees of severity, including mild, moderate, and severe forms. The group of patients included male and female adolescents and adults. Participation was voluntary and written consent was obtained from patients, or their legal protectors, before the scanning session. All experimental procedures were approved by the ethics committee of the Faculty of Medicine of the Technical University of Munich before start.

#### 2.2.3. Analysis of the Torso Scan

The software tools used to analyze the body scanner and CT data (Section 3) were implemented in Python (Python Software Foundation version 3.7.x, www.python.org) and MATLAB2018a (MathWorks, Natick, MA, USA). These tools enable the user to view, analyze, and compare 3D data from the body scanner and CT data, or X-ray images.

**Figure 2:**
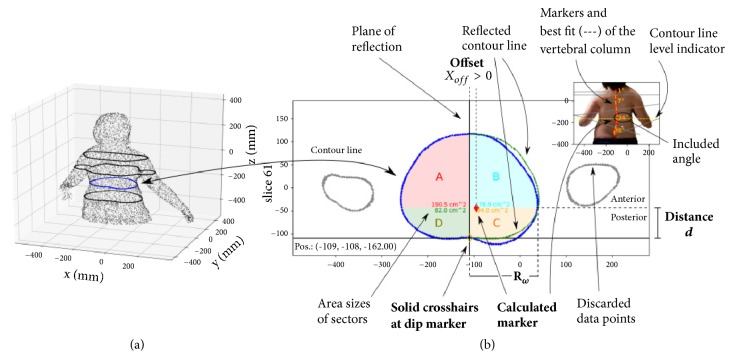
(a) 3D scattered data provided by the body scanner and corresponding contour lines at different levels along the vertical body axis. (b) Analysis of torso contour derived from a transverse plane.

In order to analyze the potential lateral deviations of the spine and the rotation of the vertebrae, the trajectory of the vertebral column has to be derived from the body scanner data. Therefore, at first, transverse sectional planes were defined on an individual basis and according to the user's criteria, at chosen levels, thicknesses and distances along the vertical body axis (Figure 2(a)). Each of these planes thus shows a contour line of the body shape (Figure 2(b)) that is then analyzed separately to find the location of the spinous processes. In practice at least ten to fifteen levels, at regular intervals along the lumbar and thoracic section, proved to be sufficient to detect the key features of the vertebral column trajectory. However, severe scoliosis in general exhibits more complex features and thus require more levels than mild scoliosis. The software makes it possible to mask data points which are not required for the analysis, for instance, the extremity or a waist band.

The final results of the analysis of each individual transverse sectional plane—the spatial positions of the vertebral column trajectory in each slice (*x*_*i*_, *y*_*i*_)—and the levels (*z*_*i*_) of the corresponding planes along the vertical axis then provide a trajectory of the vertebral column in 3D, e.g., in Cartesian coordinates (*x*_*i*_, *y*_*i*_, *z*_*i*_).

The analysis of the contour line generally starts by looking for the characteristic dip in the contour line along the median of the back, which we assume to coincide with the tip of the spinous processes. In addition we consider the inherent natural symmetry of the human body shape around the mid-sagittal plane and try to detect any deviations to that symmetry caused by the scoliosis. The software tool therefore allows the user to manually rotate and translate an axis of reflection through the contour line, along which one part of the contour line can be projected from one side to the other (Figure 2(b)). In practice the user defines a healthy and a pathological deformed side (the left and the right side, respectively, in Figure 2(b)), and projects one over the other—in Figure 2(b), for instance, the green line is the reflex of the left healthy side. The axis of reflection corresponds to the mid-sagittal line at the best match between the contour lines from the healthy and the pathological deformed side, according to the investigator's eye. A best match is achieved, to our opinion, when the reflected contour line from the healthy side adapts to the original contour line at the pathological side, where the latter shows no, or only little, pathological distortion (Figure 2(b)). The lateral position where the vertebral column intersects the transverse sectional plane can then be estimated from the symmetry line at the best adaptation of the reflected contour line to the original contour line at the pathological side. This approach is in particular helpful when no dip is clearly detectable in the contour line, or the position of the vertebral column cannot be determined otherwise. As shown in Figure 2(b), the difference between the reflected contour line from the healthy side and the pathologically deformed contour line is moreover a demonstrative visualization of the pathological deformation of the body contour at this level. An increase in this difference hence can be associated with a progression of the scoliosis. By repeating this analysis for different transverse planes, the trajectory of the vertebral column can be derived.

The curvature of the vertebral column trajectory obtained can be described mathematically by fitting a 1D polynomial to the projections of the trajectory of the vertebral column onto the coronal plane, using a nonlinear least-squares algorithm to solve the minimization problem. The degree of the polynomial is chosen by the user and based on visual inspection of the fit. A polynomial with degree one (*n* = 1) corresponds to a straight line, while *n* = 2,3, 4 correspond to a single (*C-*type), double (*S-*type), triple curved line, respectively.

*The Offset Correction.* Along the thoracic spine, in superior view, the characteristic dip in the contour line is surrounded by two symmetric, dorsally convex arches from the rib-cage. A partially rotated vertebral column, however, results in an asymmetry of the dorsally convex arches around the median, a typical indication for a scoliosis, but may have no impact on the position of the dip. In case of asymmetrically, dorsally convex arches, the vertebral body is not aligned with the spinous processes along the plane of reflection (Figure 2(b)), but is shifted laterally relative to this plane. We thus have empirically “corrected” the estimated positions of the vertebrae by individual lateral offsets to the dip at each level.

For the offset correction, the area surrounded by the contour line is separated into four sectors by crosslines—two anterior sectors (*A* and *B*) and two posterior sectors (*C* and *D*), see Figure 2(b). The lateral separation cross-line is parallel to the mid-sagittal plane through the dip at the back, or the symmetry line. The anterior-posterior separation line is perpendicular to the lateral separation line and set at a specific distance *d* to the dip (see Figure 2(b)). The parameter *d* is estimated from the analysis of transverse sectional planes at different body sections (cervical, thoracic, thoracolumbar, and lumbar) of the CT data of persons with no scoliosis (cp. Section 4).

Given the four sectors of a transverse sectional plane, the individual areas are calculated from the convex hull of each sector by the Gauss's area formula. The lateral offset correction to the initial position of the dip is then empirically estimated by

(1) $$\begin{array}{c} x_{off}=\left(\;\frac{A-B}{A+B}+\frac{C-D}{C+D}\;\right)\cdot R_{\omega }, \end{array}$$

where *A*, *B*, *C*, and *D* denote the individual areas of the four sectors and *R*_*ω*_ is the remaining width between the initial position of the dip and the intersection of the anterior-posterior separation line with the body contour at the corresponding body side. A rotation of the vertebral body, which in clinical examination often exhibits a rib hump at the back side and a corresponding flattening at the front—a characteristic asymmetry of the contour line in the transverse sectional plane—yields an offset *x*_off_ > 0 if the vertebral body is rotated towards the right side of the patient, or *x*_off_ < 0 if rotated to the left side, respectively. If the contour line is symmetric, the offset *x*_off_ = 0.

Once the “correct” positions of the vertebral bodies have been defined at each level, the curvature of the vertebral column can be derived and quantified by fitting a 1D polynomial to the projection of the trajectory of the corrected positions onto the coronal plane (as described above).

#### 2.2.4. Description of the Software

**Figure 3:**
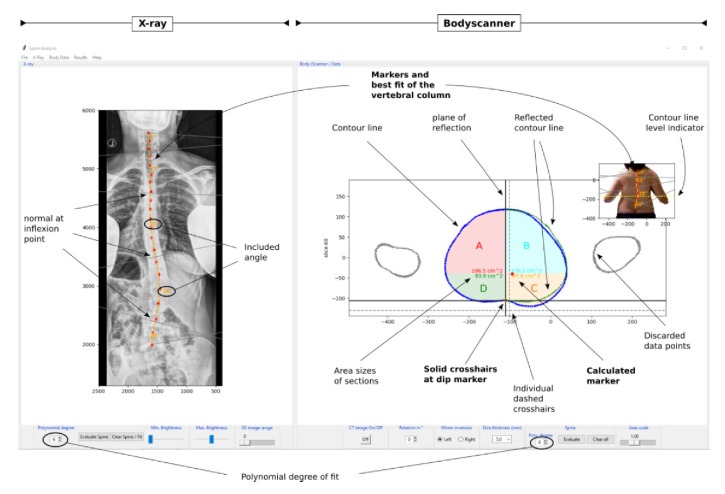
Screenshot of the analysis software tools, using an example of an adult female patient with a right convex lumbar scoliosis. The polynomial derived independently from the X-ray image (orange line in the left panel) can be compared with the equivalent polynomial derived from the body scanner data (red line in right panel).

Figure 3 shows a screenshot of the software tool developed to analyze the data. The main window is separated into two panels: one for “X-ray” data (left side) and one for “body scanner data” (right side). The X-ray panel shows the X-ray image, or a projection of the CT data onto the 2D coronal plane, in the posterior-anterior view, which can be analyzed by setting individual (red) markers along the vertebral column. The corresponding trajectory of the markers then can be evaluated by fitting a polynomial with a degree according to the users choice from *n* = 1,…, 7, using a nonlinear least-squares algorithm. The best fit is shown as an (orange) dashed line along the vertebral column. In addition, the software displays the values of the included angles in degree. These correspond to the angles between adjacent normals at the inflexion points along the vertebral column trajectory and provide an estimate of the curvatures of the spinal column. The polynomial can then be compared with an equivalent polynomial derived from the body scanner data, or previous X-ray data analysis.

The panel on the right side shows the contour line and its reflection (green dots) of a transverse sectional plane extracted from the body scanner data shown in the inset. The contour lines in the main window correspond to the transverse cut of the body shape at the level indicated by the yellow dashed line in the inset and can be changed by the user by scrolling up, or down, along the vertical body axis. The plane of reflection of the reflected contour line coincides with the vertical line of the cross hairs, at which the contour line is reflected from the healthy to the pathological side. The positions of the characteristic dip, at the back side of the contour line, and of the vertebral body can be derived as described in Section 2.2.3. These positions are marked by the orange and red diamonds, respectively, at the different heights along the spine. By setting a number of markers at different levels along the vertical body axis, the trajectory of the vertebral column in the coronal view is estimated and can be evaluated by fitting a polynomial, like it can be done on the X-ray data. The best fit and the included angles are then shown in the inset. Both polynomials—from the X-ray image and the body scanner data—can be further analyzed and compared, for instance, by the curve matching algorithm. In addition to this, the software includes the option to mark individual cross hairs at a certain position throughout all levels, for instance, the position of the vertebra prominens.

The time required to analyze a vertebral column from lumbar to thoracic section predominantly depends on the time needed to find and set the markers at the characteristic dip, or the tip of the spinous processes, respectively. An experienced orthopedic specialist takes about fifteen to thirty seconds per marker. A full vertebral column trajectory thus can be derived within three to eight minutes, depending on the clarity of the characteristic dip and the complexity of the body contour shape.

### 2.3. Results

In terms of practical feasibility of the scanning procedure with patients with limited mobility, the scanning sessions were positively rated by the participants. All patients felt comfortable with the method and could keep their position during scanning.

**Figure 4:**
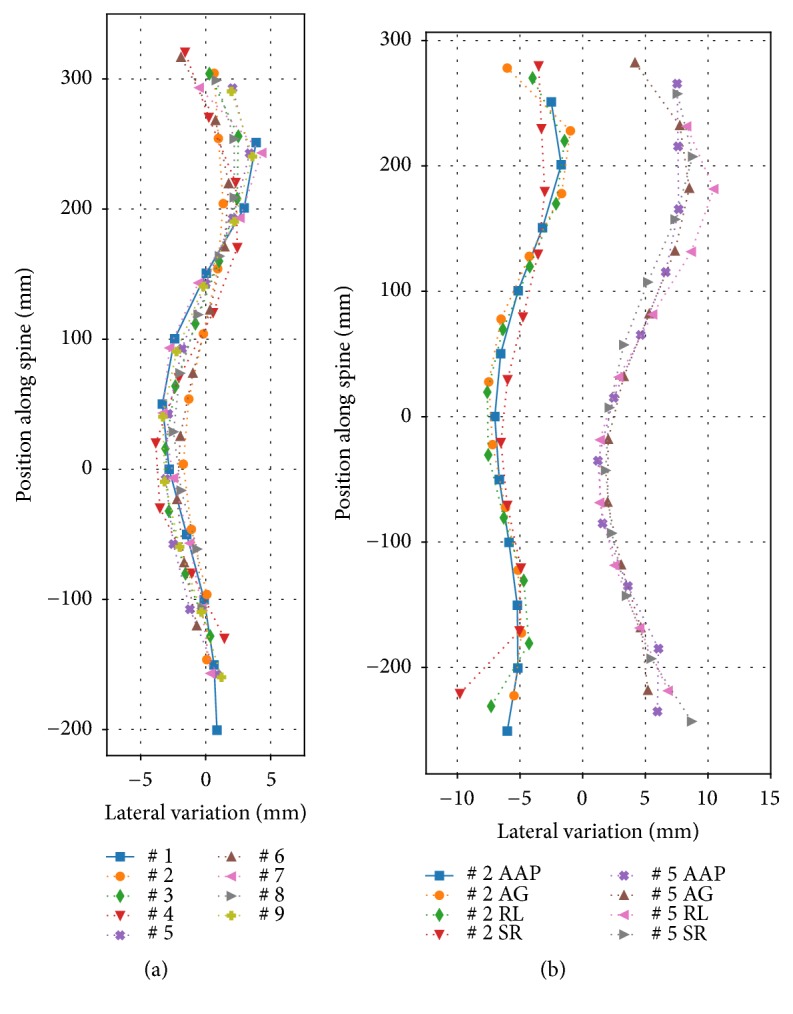
(a) Variability in repeat measurements of a person, with minor spinal deviations, all analyzed by the same observer. (Note: the abscissa (x-axis) is scaled up by a factor of 10, for better visualization.) (b) Interobserver variability in analysis of two data sets, laterally separated for better visualization, by four staff members (orthopedic specialist, neuroscientist, physicists). (Note: the abscissa is up scaled by a factor of 10.)

In order to verify the reliability of the method developed we analyzed the variability (a) in repeat measurements (intraobserver variability), as well as (b) across observers (interobserver variability). Figures 4(a) and 4(b) illustrate the variability in repeat measurements of a volunteer with a minor spinal deformation. The measurements were done at different days and times, under slightly different inclination angles of the camera, resulting in individual spinal curves from different measurements. For this participant, a clear characteristic dip could be observed, and therefore marked, along the median of the back in most body contours.  Figure 4(a) shows the spinal curves estimated from repeat measurements, all analyzed by the same observer. The variability among the spinal curves is hence due to a combination of (a) variations in repositioning of the “patient”; (b) the movements of the patient; (c) the systematic technical uncertainty (e.g., resolution of the RGB-D sensor system); and (d) the intraobserver spread in marking the dip. The latter, however, appears to be very small despite the manual adjustments. The effects of intra- and interobserver differences are shown in Figure 4(b) for four different observers, each analyzing two different data sets (laterally separated for better visualization). Both figures show small variances of less than 5 mm in lateral deviation of the spinal curves. The intraobserver variations are slightly larger than the interobserver variations, but small for the amount of manual adjustments involved in the analysis methods in either case. The larger intraobserver variations are an indication that the repositioning, the patient's movements, and the systematic technical uncertainty dominate over the interobserver variations. Further technical and methodical improvements are therefore being currently addressed.

## 3. Validation of the Analysis Method

### 3.1. Motivation

Given the analysis methods described in the previous section (Section 2), the next step was to verify if they could deliver reasonable estimates of the trajectory of the vertebral column. Therefore we have analyzed a series of torso contour data extracted from CT images. Thereby we could directly compare the trajectory of the vertebral column, derived from the contour line analysis, with the “real” positions of the vertebral bodies extracted directly from the CT images.

### 3.2. Methods

#### 3.2.1. CT Data

In total we have analyzed twenty-five sets of CT data, provided by the radiology department of the Klinikum rechts der Isar of the Technical University of Munich for verification of our analysis method. These data sets were not collected purposely for this study, but were selected from the pool of images of the patients attending the department for other reasons but who incidentally showed a malposition of the spine. The set of data comprises patients between 18 and 86 years of age, of both genders (12 female and 13 male) and with different types of scoliosis and spinal malpositions. The CT images were analyzed by a senior orthopedic specialist, who classified seven out of the twenty-five cases as thoracic scoliosis, eleven as lumbar, four as thoracic-lumbar, and three as combined scoliosis. Data were anonymous and all procedures were approved by the ethics committee of the Faculty of Medicine of the Technical University of Munich before starting the study.

#### 3.2.2. Analysis of the CT Data

**Figure 5:**
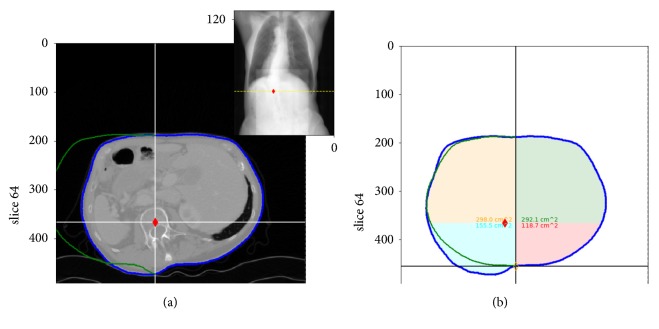
Two views of a CT image. (a) Real view of CT image for verification of vertebral body position. The red diamond marks the vertebral body position. Inset shows “CT to X-ray” image, extracted from CT data. (b) Contour line extracted from the CT data with the estimated vertebral body position marked by a red diamond.

The software tools, introduced in the previous section (Section 2.2.4), also provide the option to analyze CT data and the projection of the CT data onto the coronal plane. In detail, the CT data were analyzed in different ways:

- From the 2D projection of CT data onto the coronal plane (“CT to X-ray”), like an X-ray image (inset of Figure 5(a)). The “CT to X-ray” images in the posterior-anterior view were visually analyzed, and (red) markers were manually set at the positions of the vertebral bodies along the vertebral column.
- By individually extracting transverse sectional contour lines from the CT data without showing the inner structure of the body, similar to the output of the body scanner, and then setting markers at the characteristic dip on the contour at the back side (Figure 5(b)).
- By individually setting (red) markers at the center of the vertebral bodies at the corresponding transverse slices of the CT data (Figure 5(a)).

The “CT to X-ray” images were extracted because diagnosis of scoliosis is usually based on 2D X-ray images and clinical examination. Furthermore, in the future the spinal curvature derived from the 3D surface scan provided by the body scanner will be compared with the equivalent curvature obtained from the X-ray image. So it was important to validate our analysis method with an X-ray-like view. In either way, the trajectory of the vertebral column given by the positions of the markers as a function of the corresponding transverse slices was always analyzed by fitting a 1D polynomial to it (Section 2.2.3).

### 3.3. Verification Using CT Data

Verification was based on the following properties.

#### 3.3.1. Trajectory of the Vertebral Column

In complement to the posterior-anterior view of the torso, shown in the inset of Figure 5(a), the transverse sectional contour line was overlaid with the corresponding CT image for an immediate verification of the estimated vertebral body position, marked by the red diamond, relative to its exact position. The trajectory of a vertebral column was calculated from three sources:

- From the positions of the vertebral bodies obtained by visual inspection of the “CT to X-ray” images – below denoted as SP1 (inset of Figure 5(a)).
- From the positions of the characteristic dip on the CT-derived torso contours, with our method as described in Section 2 – SP2 (Figure 5(b)).
- From the exact positions of the center of the vertebral bodies in the transverse sectional CT images – SP3 (Figure 5(a)).

The trajectories derived from the vertebral body positions (SP3) provide the most accurate results and constitute the reference trajectories. Hence, comparison of the trajectories was done with SP3 vs. SP1 and with SP3 vs. SP2.

**Figure 6:**
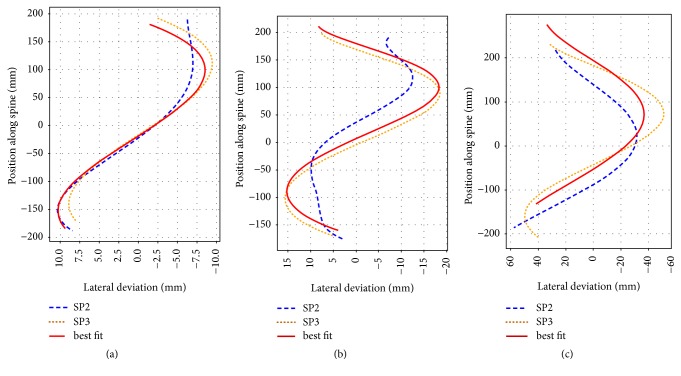
Results of the fit to the spinal trajectory obtained from body contour line analysis and vertebral body position analysis. Dashed line: trajectory from body contour line analysis (SP2), dotted line: trajectory from vertebral body position analysis (SP3); solid line is the best fit line of SP2 on to SP3. (a) is an example of mild deviation, (b) moderate deviation, (c) strong deviation. (Note the different scaling of the abscissa for better visualization.)

In the following we classified the lateral deviation of spine from the CT scanned images, obtained with the developed tools, as mild when the deviation was ≤10  *mm*, moderate when the deviation was in between 10 − 20  *mm* or strong when ≥20  *mm*. Examples of lateral deviations derived from the vertebral body positions (SP3) and from the positions of the dips at the body contour lines (SP2) are presented in Figures 6(a)–6(c) for three different cases: a small lateral deviation of ~10  *mm* (Figure 6(a)), a moderate deviation of ~20  *mm* (Figure 6(b)), and severe deviation of ~40  *mm* (Figure 6(c)).  Figure 6 illustrates visually that curves are more alike (better match) for the case of small deviation than for the other cases.

A standard nonlinear least-squares algorithm was then used to match the three trajectories. The fitting variables applied to the center point of the curve were an unconstrained translation in the coronal plane and a rotation around the normal at the center point in the coronal plane, limited to an angular range of −45° < *α* < +45° (to avoid a match when one curve is upside-down relative to the other).

#### 3.3.2. Optimality Values

**Figure 7:**
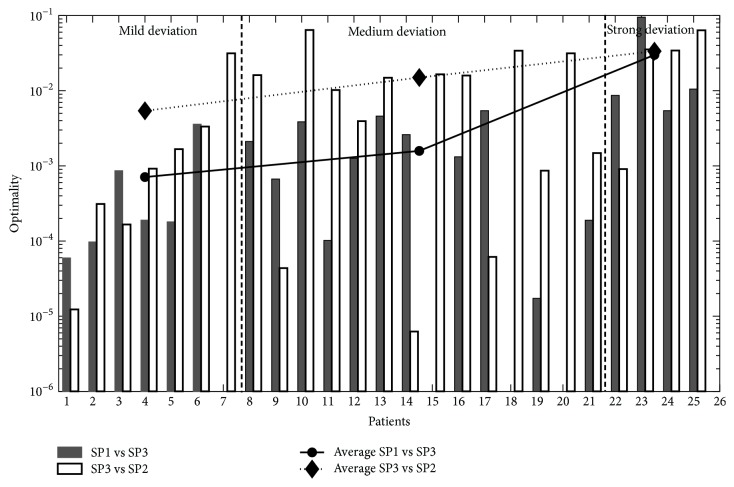
Optimality values (in logarithmic scale) are plotted for each CT data set. Filled bar indicates optimality values between the trajectories obtained from 2D X-ray image analysis and vertebral body analysis (SP1 vs. SP3), open bar indicates optimality values between the trajectories obtained from vertebral body analysis and the body contour line analysis (SP3 vs. SP2). Circle with solid line is the average of optimality between the SP1 vs. SP2 sets of trajectories and diamond with dotted line is the average of optimality between the SP3 and SP2 sets of trajectories. Missing bars correspond to cases for which the quality of the 3D surface scan was not good enough for a clear analysis.

The quality of the match was quantified by an optimality parameter, which is related to the corresponding residuals. The lower the optimality parameter is, the better the match between the two trajectories.  Figure 7 shows the optimality values for each patient obtained from matching the trajectories derived from “CT to X-ray” images (SP1) with trajectories obtained directly from the CT scans (SP3), SP1 vs. SP3 (left filled bars); right bars (open bars) represent optimality values between the trajectories obtained directly from CT scans and from the contour line analysis (i.e., between SP3 vs. SP2). The figure is divided into three portions, separated by vertical dashed lines: the left part shows the optimality values obtained for cases of small vertebral malpositions and mild scoliosis, the middle part for cases of moderate scoliosis and the right for cases of severe scoliosis. The two lines show the mean of the optimality values in each of these parts. The gradual increase of the mean optimality value with respect to the severity of scoliosis indicates an increasing mismatch between the trajectories with increasing severity of scoliosis.

#### 3.3.3. Locating Vertebral Body and Spinous Processes

**Figure 8:**
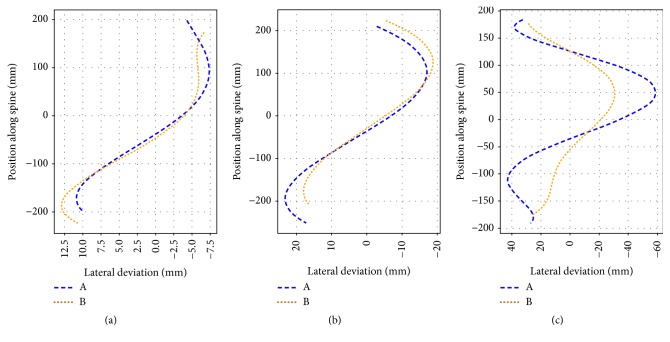
Lateral deviation analysis locating vertebral body and spinous processes positions. “A” indicates the trajectory locating vertebral body positions, “B” indicates the trajectory locating spinous processes positions. Figure (a) is an example for mild deviation, (b) is an example for moderate deviation, and (c) is an example for strong deviation.

In addition to comparing the lateral deviation derived from the location of the vertebral bodies (Figure 6) to that obtained directly from the CT scans (SP3), we also compared that deviation with the one derived from the locations of the contour dips (for the same case). If these two deviations are similar, then the lateral deviation obtained from the trajectory derived from the contour dips, which is easier to estimate than that based on the vertebral body positions, provides the necessary information to estimate the degree of lateral deviation or scoliosis. Hence, the deviation derived from the positions of the vertebral bodies was compared with that derived from the positions of the spinous processes. The latter positions are basically those of the contour dips at different levels in height. Figures 8(a)–8(c) show the lateral deviations obtained for a case of mild, moderate, and strong deviation, respectively. Visual inspection shows that there is a better match for cases of mild and moderate degree of scoliosis than for the case of severe scoliosis. In the latter case the difference in lateral deviation varied up to 30  *mm* (Figure 8(c)). The larger discrepancy for the case of strong scoliosis indicates that there is a strong internal twist of the vertebral bodies relative to the tips of the spinous processes. On the other hand, the better matches observed for mild and moderate lateral deviations indicate that the internal rotation is smaller in those cases.

## 4. Additional Results

### 4.1. Angular Rotation of Vertebral Body with respect to Spinous Processes

**Figure 9:**
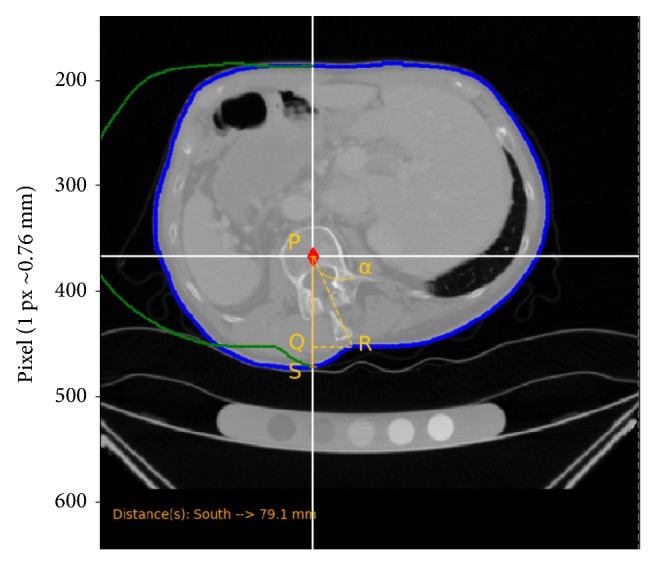
Estimate of the angular rotation of the vertebral body with respect to its spinous processes and of the shift (QR) of the spinous processes position on the contour line.

In order to get an idea about the rotation of the vertebral body with respect to the tip of the spinous processes, a quantitative analysis was made, as shown in Figure 9. The position of the vertebral body (P), the position of the dip on the contour line (R), and the vertical distance of vertebral body to the contour line (PS) were marked with the developed tool. Then simple trigonometric implementations were used to calculate the angle (*α*) formed by the vertices *QPR* and the distance QR (see Figure 9). In the case shown, a rotation of ~23° of the vertebral body with respect to its spinous processes produced a linear shift (QR) of the characteristic dip of ~25  *mm*.

### 4.2. Distance of Vertebral Body to the Skin

We measured the distance of the vertebral body, perpendicular to the back surface of the skin (*δ*), directly from the CT scans. The aim was to see if there is a typical relative position of the vertebral body to the back skin surface, distinguishing the different types and severity degrees of scoliosis. For each individual the distance was obtained at several transverse sections along the column. In case of no scoliosis, this distance (*δ*) corresponds to the distance *d* shown in Figure 2(b), and, for the case shown in Figure 9, to the distance *PS*. To account for the effect of body size and individual constitution on the absolute distance, these values were furthermore normalized using the formula:

(2) $$\begin{array}{c} \Delta \bar{\delta }=\frac{\delta_{i}-\bar{\delta }}{\bar{\delta }},\;where\;\;\bar{\delta }=\frac{\sum_{i=1}^{n}\delta_{i}}{n}, \end{array}$$

*δ*_*i*_ denotes the individual distance and *n* the number of data points, equal to the number of transverse planes analyzed.

**Figure 10:**
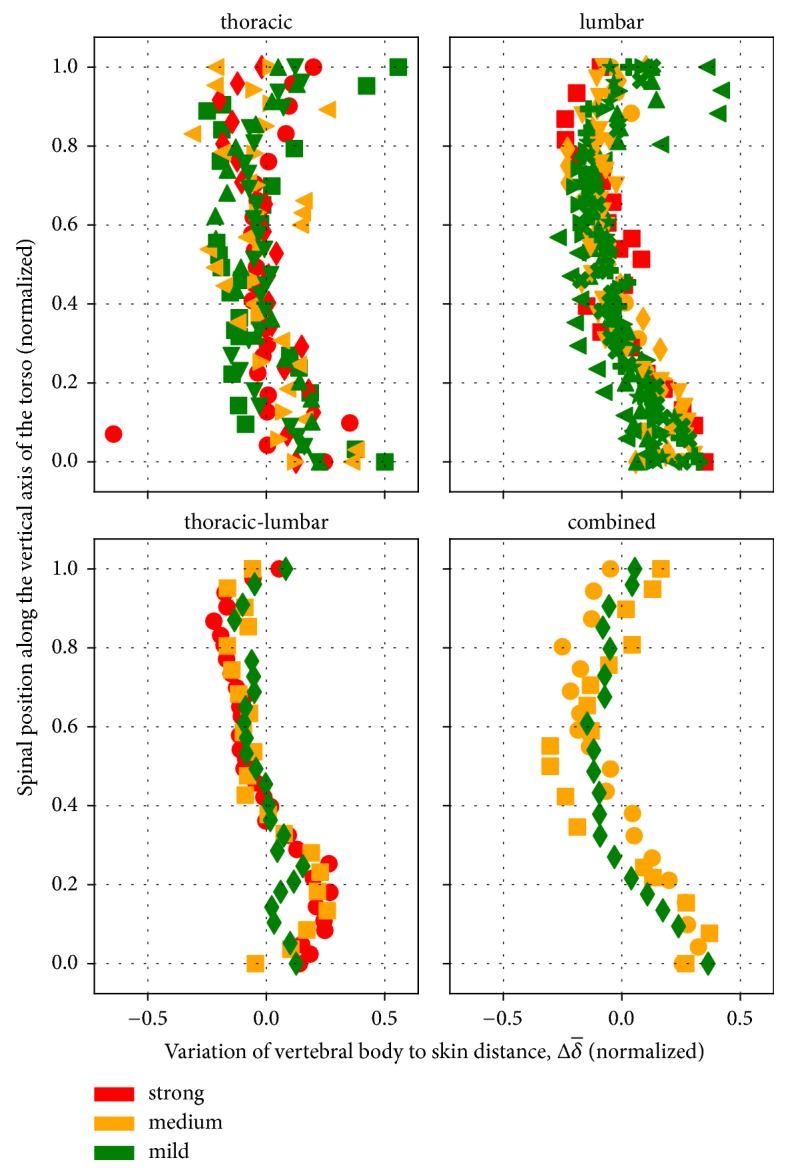
Variation of the individual mean of vertebral body to skin distances, for different types of scoliosis (green symbols represent small deviations, yellow moderate deviations, and red strong deviations). Each panel represents the classification of scoliosis according to the vertebral column region: thoracic, lumbar, thoracic-lumbar, and combined scoliosis. The lengths of the vertebral columns were normalized from 0 (level of the iliac crest) to 1 (level of the vertebra prominens) to account for interindividual differences in length.

Figure 10 shows the relative deviations of vertebral body to skin distances ($\Delta \bar{\delta }$, in equation (2)) from the individual mean, for all the available CT data sets, according to the severity of scoliosis (illustrated by different colors) and its section along the vertebral column. The set of available data does not show any clear feature in the way *δ* changes with level. All types of scoliosis show a similar trend, with slightly larger distances occurring in the middle-upper and lower parts of the torso. The groups of thoracic and lumbar scoliosis show a few larger distances relative to the other groups, especially on the upper levels. The other groups show a more homogeneous trend, however, this may simply be due to the fact that the number of patients is also smaller in these, relative to the group with thoracic scoliosis. Extending the analysis to more cases may reveal characteristic features for each group. Overall, Figure 10 shows that *δ* does not change substantially with level and, therefore, our initial assumption of a constant intraindividual vertebral body to skin distance (*d* in Figure 2(b); see also offset correction part in Section 2.2.3) is reasonable. Nevertheless, the access in the future to accurate values of *δ* with level will help to improve the estimation of the vertebral body position within each body contour.

### 4.3. Different Types of Contour Shapes

**Figure 11:**
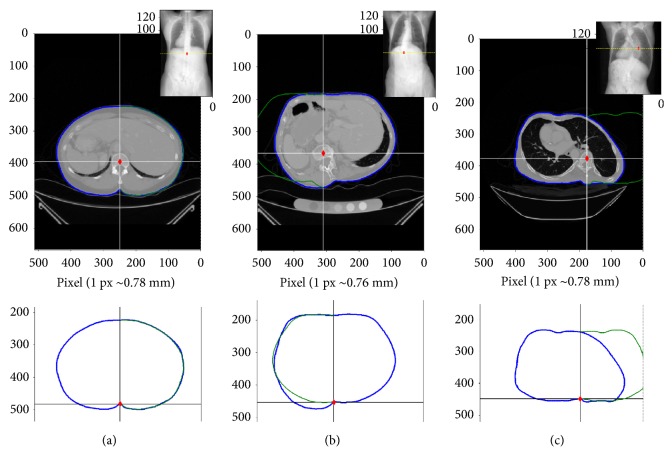
Examples of typical body contour shapes extracted from the CT scan images. (a) Body contour showing a symmetric shape, associated with a mild spinal deviation. (b) Example of a contour shape associated with a moderate deviation and (c) example case associated with a strong deviation. The upper panels show distinct vertebral positions on the transverse CT view of the images. The lower panels show the corresponding contour lines with the characteristic dips extracted with the developed software. Insets of upper panels show 2D “CT to X-ray” views of CT scanned images.

Three distinct types of torso contour shapes in the transverse section could be distinguished among the twenty-five CT scans, as illustrated by three example cases in Figure 11.  Figure 11(a) is one type of transverse contour shape with a prominent contour dip, associated with a mild lateral spinal deviation. Figures 11(b) and 11(c) are other two types of contour shapes, which are associated with moderate and strong deviations of the spine, respectively, due to the rotation of the vertebral bodies. A change in contour shape thus indicates a clear change in spinal curvature.

## 5. Discussion

In the current work we present an ionizing radiation-free method, to estimate spinal deviations, based on the analysis of 3D surface scans of the torso captured with a body scanner. An analysis method and associated software were developed with the aim of estimating the position of the vertebral body and its rotation with respect to spinous processes from 3D surface scans. The method presented was validated by applying it to a series of twenty-five CT scan images.

Visual analysis of lateral spinal deviations obtained from different views of CT images showed a good match for cases of small lateral deviations of the spine (Figure 6), as confirmed by the lower optimality values (Figure 7) relative to more severe cases. Similar results were obtained when comparing the deviation derived from the locations of spinous processes with that from the locations of the vertebral bodies (Figure 8). Also here a better match was obtained for mild to moderate cases of scoliosis. The partial mismatch for severe cases seems to be related to the rotation of vertebral bodies. Indeed, in adolescent scoliosis a rapid growth of vertebral bodies is observed, that is not accompanied by a similar growth of the dorsal parts [19]. This imbalance leads to malpositioning of the vertebral bodies, including rotation around the vertical body axis and lateral tilt. This becomes, in turn, reflected in an altered shape of the torso contour (Figure 11), without affecting much the position of the spinous process (Figure 11). Hence, the malposition or rotation of the vertebral body is likely the main cause for the partial mismatch of estimations obtained for the severe cases of scoliosis, despite the offset correction (*x*_off_) applied to obtain a more realistic position of the vertebral body. The correction procedure, which produced good results for mild and moderate scoliosis, thus needs to be further improved in cases of severe scoliosis.

The scanning method shares with other noninvasive methods, such as raster stereography [13, 20–23], the advantage that it is ionizing radiation-free and has short scanning times. The latter tends to reduce the effects of artifacts on the 3D surface scans due to body movement during scanning. This is especially relevant when examining children or patients who cannot stand for long. Another advantage of the method is that it does not require setting markers on the torso of the patient. Since the 3D body profile scan is derived from a “continuous film”, taken at a full circle around the body, the present method delivers high resolution images of the body surface from any perspective in the transverse plane and requires less interpolation, because there are fewer hidden areas than when reconstruction is made from a smaller number of images at static positions. Hidden areas may however occur in case of females with heavy breasts, with torso contours in areas under the breast not being well defined, even with the present system. A severe scoliosis will produce in these cases an asymmetry in the shape of the contour in these areas, which will affect the estimations made. Although we have not encountered such cases so far, this issue will be addressed in future work, potentially with help of subject specific mathematical modeling of the rib-cage.

The method presented gives, similarly to other surface topography methods, the possibility of representing and analyzing scoliosis and estimating, in an alternative way, the degree of rotation of the vertebral body at different levels of the torso contributing to the scoliosis. In case of the Moiré method [11, 12, 24] such rotation is reflected in deformities of the surface topography and consequently on changes in the Moiré patterns that are generally quantified by the Suzuki Hump Sum parameter [5, 25]. Also rasterstereography can provide similar measures of rotation from the detection and characterization of deformities in the surface topography [5]. Other studies, however, found weak correlation between the spinal and surface deformities especially in younger children, for which remarkable thoracic asymmetries were observed without any detectable spinal deformity in the radiographs [26, 27]. Hence, and especially in cases of idiopathic scoliosis, the exact etiology of which remains unclear though being generally believed to be multifactorial [28, 29], any surface-based methods should be used cautiously and only after a thorough clinical examination.

The current, as well as other nonionizing radiation based methods, however cannot replace the conventional methods (e.g., radiography) in the medical assessment of scoliosis. Especially in cases of severe or advanced scoliosis, decisions will continue to be based on X-ray and MRI, if required. These are necessary in case surgical intervention is considered, as exact anatomical information about the vertebral column is essential. Assessment of scoliosis with the methods presented here is rather envisaged to complement conventional assessments, particularly by helping to trace the course of change in scoliosis.

**Figure 12:**
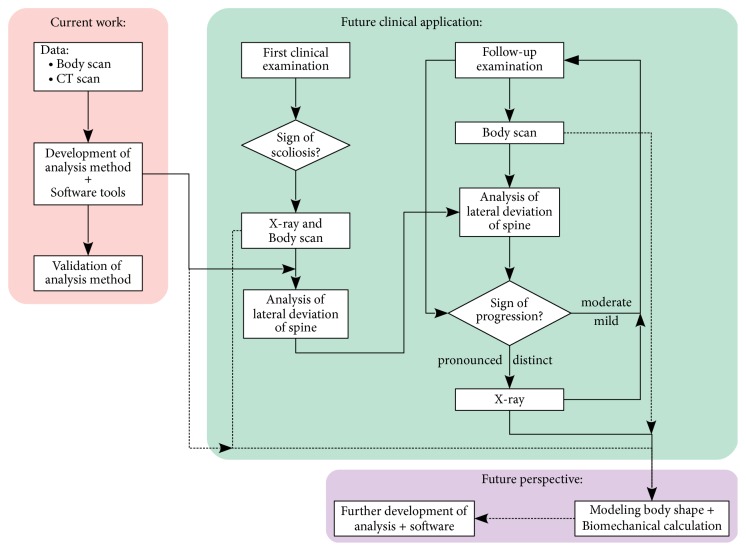
Schematic representation of the aim of the present work and of planned developments and application.

The work here presented constitutes a first step in the development of a method that can support the clinical evaluation of progression of scoliosis (Figure 12). This support is, at face of the current results, closer to be applicable in cases of mild and moderate scoliosis. A good agreement was also obtained between the estimates of spine trajectories derived from the body scanner and X-ray images. An X-ray image will generally be required for the first appointment with the orthopedic specialist. Later on, a decision on whether an X-ray is required, or not, will always critically depend on the clinical examination done by the specialist: if during follow-ups a progression of the scoliosis is clinically identified, an X-ray examination will always be needed. But if there are no detectable changes in the status of the scoliosis, then follow-ups may be supported by 3D body scans. Hence, if two 3D surface scans taken at different times (for example, at the first and second medical examination) are not significantly different, this may support a decision against doing another X-ray in that moment. Further, for mild and moderate cases of scoliosis small changes may occur that may not be detectable in a clinical examination, but that may be better identified and characterized in a 3D scan. However, further developments are still needed to reach this stage. These developments need moreover to be able to discriminate changes due to progression of scoliosis from other sources, such as growth. In fact, especially in cases of idiopathic scoliosis, growth has a significant effect on the correlation between the progression of scoliosis and the shape of the torso contour [26]. Changes due to different body positions in follow-up scans are currently minimized by complementary camera and software tools that help to set the patient in the same position.

Assessment of the spinal curvature from 3D scans of the torso may also be helpful in following the fast progression in cases of severe scoliosis, in addition to X-rays. A reliable procedure that can be employed in these cases of advanced status of scoliosis and fast progression requires still gathering and analyzing more 3D surface scans in the course of follow-ups, to improve the analysis software. Evaluation of progression of scoliosis will benefit, in future developments, not only from continuing the analysis of new cases, but also from the biomechanical modeling of the rib-cage.

The results presented clearly support the reliability of the spinal trajectories derived from body scanner surface scans. The method and software presented are moreover applicable to any point cloud that represents the body surface in 3D and are thus not confined to a specific sensor system. Nevertheless, a final validation of the methods will need to be based on the comparison of the estimates from the body scanner with trajectories extracted from CT images, both collected from the same person (that was not the case here, where CT images and body scanner surface scans had been collected from different groups of patients). Also the effects of growth on progression of torso shape and scoliosis will need to be addressed in future work.

## 6. Conclusions

- Software tools were developed to estimate the spinal curves from analysis of 3D surface scans of the torso.
- Validation of the tools was done by using them to analyze twenty-five CT scans of adults who had a malposition of the spine and/or a scoliosis.
- The methods presented delivered good estimates of the vertebral column trajectory in cases of mild and moderate scoliosis. Severe cases of scoliosis require further developments.
- The analysis methods presented are not intended to replace the conventional radiography-based methods used to assess a scoliosis. The method is to give supplementary information in cases of mild and moderate deviations, for which progression can still occur, but which may not justify an X-ray examination.

## Data Availability

The clinical data used to support the findings of this study have not been made available in accordance with the protection of data privacy.
